# Predictors of International Entrepreneurial Intention Among Young Adults: Social Cognitive Theory

**DOI:** 10.3389/fpsyg.2022.894717

**Published:** 2022-06-10

**Authors:** Milad T. Jannesari

**Affiliations:** School of Business, Zhejiang University City College, Hangzhou, China

**Keywords:** international entrepreneurial intention, parent socioeconomic status, sense of power, cultural intelligence, work experience

## Abstract

This study investigated the psychological mechanism by which parents’ socioeconomic status, including income and social class, influences the international entrepreneurship intentions of young adults. Two datasets, self-reported (survey) and objective, were collected from 372 undergraduate students across 19 universities in China. Parents’ income and social class had a positive effect on international entrepreneurship intentions. Sense of power and motivational cultural intelligence (CQ) played mediating roles in this relationship, and work experience moderated this relationship. The mediation tests revealed that sense of power and motivational CQ comprise a serial mediation process, in that order. The effect of motivational CQ on international entrepreneurship intentions was strengthened by young adults’ work experience. We identified the underlying mechanism and moderator of the relationship between socioeconomic factors and international entrepreneurship intentions.

## Introduction

With the accelerating globalization of the world economy, scholarly attention to international entrepreneurship (IE) has increased rapidly over the past decade (Ahsan and Fernhaber, 2019). Specifically, scholars [e.g., Yao et al. (2020)] have developed a strong interest in the emerging IE mindset among young adult generation. They believe that the transition from school-to-work is an important period for understanding successful IE (Middermann, 2020). This also concerns the major societal goal of educating the next wave of IE in order to improve the international entrepreneurial capabilities to meet the global challenges of the twenty-first century (Zucchella, 2021).

Many previous studies have attempted to identify factors that affect entrepreneurial intentions, such as education (Liñán et al., 2011), contextual factors (Turker and Sonmez Selçuk, 2009), and personality traits (de Janasz et al., 2007). However, few studies have investigated factors that affect IE intention. To date, scholars have only recognized personality-related determinants of IE intention (e.g., proactive personality, personal attitude), which has left the role of contextual and psychological predictors largely unexplored (Jie and Harms, 2017; Middermann, 2020). The present study answers the call for research on IE intention [e.g., Jie and Harms (2017)] by examining the following research question: What factors nudge young adults toward IE as a career path?

International entrepreneurship is generally understood to involve the discovery, enactment, evaluation, and exploitation of business opportunities that span national borders and involve the global environment (Chandra and Coviello, 2010). According to Jomah (2018), interest in starting a new business depends on various factors, such as psychological, social, academic, and family issues that influence entrepreneurial intention early on, i.e., during college education or even earlier. As a contextual predictor, parents’ socioeconomic status (SES) comprises a key external factor that can develop or hinder one’s entrepreneurial interest and, thereby, influence post-graduation career choices (Jomah, 2018). The present study employed the Social Cognitive Theory of Social Class (Kraus and Stephens, 2012) to explore whether young adults from upper or middle social classes are more likely to pursue IE than their lower social class counterparts. Social Cognitive Theory of Social Class views parents’ SES as a context rooted in both the material substance of social life (income and social class) and the individual’s construal of his or her parents’ class rank (Dubois et al., 2015). This theory highlights the material dimension as a core aspect of how an individual thinks of the self, reaches self-clarification, and develops a personal orientation that will influence decision making across various aspects of life, including career choices.

As a second contribution, the present study answers the repeated calls for identifying the psychological process that leads to IE intention (Jie and Harms, 2017; Yao et al., 2020). While, sense of power and cultural intelligence (CQ) are seemingly important for international entrepreneurs’ success, these factors have not been widely studied in conjunction with IE intention. On the one hand, the role of CQ in developing entrepreneurs’ cognitive ability to adapt to the business environment has been recognized by Social Cognitive Theory (Bandura, 1989). On the other hand, sense of power (i.e., feeling empowered) has been linked to an individual’s socioeconomic resources, as well as the degree of his or her CQ (Caputo et al., 2019; Murphy et al., 2019). This paper conceptualized and empirically tested sense of power and CQ as the potential psychological mechanisms underlying IE intention.

**FIGURE 1:**
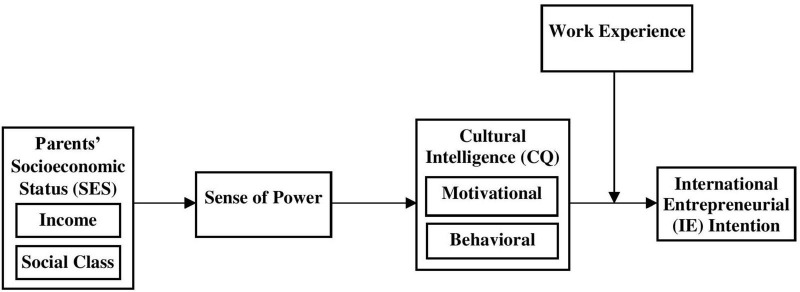
The conceptual model.

In the next section, we argue that the association between parents’ SES and an individual’s IE intention might be better understood by considering how SES influences sense of power and CQ. We further explored the moderating roles of work experience, as shown in our conceptual model (Figure 1).

## Literature Review

Because of the continuing expansion of higher education in China, the number of college graduates has grown dramatically since 1999 (Shi and Xing, 2010). Consequently, the employment pressure on college students is more intense than that of past college graduates. Therefore, entrepreneurship and international entrepreneurship can play essential roles in creating employment opportunities for college graduates (Hu and Ye, 2017). A new policy was developed in China by the Ministry of Education, called the National Graduate Entrepreneurship Initiative, with the goal of assisting graduates in starting up small businesses (Millman and Li, 2017). However, the proportion of college graduates in China who become entrepreneurs remains low (Yao et al., 2020; Sun et al., 2022). To encourage college students to overcome psychological barriers and become more entrepreneurial, it is necessary to identify factors that might influence this ability.

Indeed, a critical feature of today’s global economy is the growing role of young adults in new entrepreneurial ventures (Mehtap et al., 2017). With the introduction of the Belt and Road Initiative in 2013, China is now producing more young entrepreneurs than ever before (Li, 2018). Furthermore, higher education institutions have started to raise awareness about IE opportunities among Chinese students, and imbue them with IE intentions (Li, 2018). Combined, these factors mean that IE is now a far more accessible dream for young Chinese adults. Furthermore, up-and-coming Chinese nationals who seek international business opportunities are not as eager to inherit and manage family businesses as their parents once were (Gunessee and Hu, 2021).

## Theoretical Background and Hypotheses

### Parents’ Socioeconomic Status, Sense of Power, and International Entrepreneurship Intention

In general, people respond to their career choices according to what they consider possible and how they envision their future career (Jannesari and Sullivan, 2019, 2021). In one study, Kraus and Keltner (2013) investigated the link between social cognitive tendencies and SES. The authors found that people from different SESs develop different social cognitive tendencies that explain substantial differences in perceptions about career-related prospects. High-SES families are thought to provide family members with more educational opportunities and social capital (Caroleo and Pastore, 2012; Kraus et al., 2012), thus preparing them for superior career choices. By contrast, it is thought that low-SES families tend to be short in essential resources and prone to poverty-induced psychological stress, which hinders career ambitions (Kraus et al., 2012).

In this study, we employed Social Cognitive Theory of Social Class to explain IE intention according to SES differences between families. SES comprises parents’ material resources (i.e., income) and social class rank, and gives rise to interpersonal differences in terms of social cognitive tendencies, which in turn influence behavioral intentions. Social class is defined as an individual’s subjectively experienced social class ranking of the self in relation to others in society (Adler et al., 2000). High-SES families are thought to provide family members with more educational opportunities (Caroleo and Pastore, 2012; Kraus and Stephens, 2012), thus preparing them for superior career choices. In contrast, low-SES families are thought to lack essential resources and are prone to poverty-induced psychological stress, which hinders career ambition (Kraus and Stephens, 2012). Multiple studies have found that family SES affects college students’ attitudes, academic performance, and career prospects (e.g., increases their self-efficacy) (Blustein et al., 2002; Turner and Lapan, 2003). According to one study, students from low-SES families have lower self-esteem and fewer academic accomplishments than those from high-SES backgrounds (Feng and Guo, 2017). Specifically, we compared how young adults with upper- vs. lower-class parents perceive themselves and the social world, and to what extent they form IE intention.

Furthermore, students with high SES have a better opportunity to develop their cultural competence and resources than students from lower SES. As a result, SES makes it easier to find and allocate resources needed to meet the demands imposed on students. As a result, students with high SES may be better able to identify opportunities that could enrich their cultural knowledge and increase their understanding of cultural differences, which leads to further interest in becoming an entrepreneur overseas. Thus, SES is expected to increase student’s IE intention. Therefore, we formulate the following hypotheses:

H1: Parents’ (a) income and (b) social class are associated with young adults’ IE intention.

While, these studies underscore the importance of parents’ role modeling and SES, they do not shed light on the psychological process that leads to IE intention. To this aim, we extended these studies by delineating the effect of two serially related mediators. Kraus and Stephens (2012) have argued that class-related social cognitive tendencies result in differences in self-conception in terms of how much control one has over one’s life and causal attributions made about other people’s actions and the social environment. Since perceived control reflects the perceived degree of power held (Johnston et al., 2018), individuals from high-SES families are expected to enjoy a greater sense of power, both objectively (i.e., the depth and breadth of resources parents avail to them) and subjectively (the lived experience of having the power to initiate and accomplish tasks). People with greater resources exhibit greater feelings of powerfulness (Kraus et al., 2012).

Power is essential to both the concept and practice of entrepreneurship. In fact, many view entrepreneurship as a goal-oriented accumulation and deployment of resources that can be used to yield the power to achieve goals [e.g., Begley and Boyd (1987)]. Consequently, individuals who have access to their high-SES parents’ resources tend to yield more power relative to their low-SES family counterparts (Kraus et al., 2012). As an asymmetric control over resources in social relationships (Magee and Galinsky, 2008), sense of power can expand an individual’s career horizon and open his or her perception about self-employment possibilities. Therefore, parents’ income and social class could influence one’s sense of power and, by extension, IE intention. We therefore made the following hypothesis:

H2: Parents’ (a) income and (b) social class are associated with young adults’ IE intention indirectly via sense of power.

### Sense of Power, Cultural Intelligence, and International Entrepreneurship Intention

Transportation, communication, and information technologies have taken globalization and intercultural diversity to the next level; thus the next generation faces abundant and exciting international business opportunities (Rasul et al., 2017). With increased cross-border interactions, as well as increased diversity within a country’s political borders, comes a plethora of new challenges and learning opportunities; furthermore, entrepreneurs may wish to replace rigid and culturally insensitive ways of doing business with flexible and cross-culturally affirmative ways. These capabilities fit with what has been labeled CQ (Earley and Peterson, 2004).

According to the social cognitive theory, the only way to develop social cognition is to engage in social interactions and continue to invest in social ties (Nakhaie and Kazemipur, 2013). It is possible that social interactions can be converted into valuable resources through CQ to assist individuals in accumulating resources. CQ may enhance an individual’s understanding of how information can be integrated and applied to develop new resources. This allows resources to be pooled, and encourages individuals to invest in resources for future use (Adler and Kwon, 2002). The present study suggests that CQ provides a mechanism that facilitates students’ ability to grasp new knowledge and experiences to resolve cross-cultural problems. Students can improve their CQ by participating in various cultural and educational activities involving cross-cultural interactions (Earley and Ang, 2003). This study concludes that entrepreneurs can enhance their cultural competency by cultivating social contact with host country residents. For example, a previous study reported that contact with cross-cultural groups can help individuals develop a positive attitude toward a new culture and motivate them to adapt to the cultural norms and traditions of the host country (Pettigrew et al., 2011).

Higher CQ has been associated with a stronger interest and performance in cross-cultural careers (Rasul et al., 2017). Those with higher motivational and behavioral CQs are reported to cope better with uncertain situations (Zimmerman and Paulsen, 1995). Indeed, from an information processing perspective, a higher CQ level is associated with a greater capacity to store and categorize new experiences. CQ can reduce the uncertainty that is associated with IE activities, thereby increasing the intention to engage in IE. Prior research suggested that individuals with a high CQ are more likely to excel in entrepreneurial activities (Ang et al., 2007). According to Social Cognitive Theory, one’s ability to successfully complete a task can be enhanced by observing others completing the task in the context of social interactions and experiences (Zimmerman and Paulsen, 1995).

Cultural intelligence is also associated with sense of power. When entrepreneurs feel empowered, they are more confident when initiating cross-cultural interactions and relationships (Dovidio et al., 1988), holding culturally appropriate eye contact (Henley, 1973), and recommending less complex cognitive approaches to problem solving (Gruenfeld, 1995). Together, this evidence suggests that motivational and behavioral CQ underlie the links between sense of power and IE intention. Individuals who feel empowered tend to seek and utilize more information about foreign cultures, exhibit more confidence in their judgments, and appear relatively bolder when it comes to IE opportunities (Yao et al., 2020). As such, CQ may serve as a mechanism through which sense of power influences IE intention.

H3: Sense of power is associated with young adults’ IE intention via (a) motivational CQ and (b) behavioral CQ.

### The Moderating Effect of Work Experience

Social Cognitive Theory recognizes that individual actions, thoughts, and behaviors are impacted by a combination of observation and direct experience (Bandura, 1989). The present study considered work experience as an element of workplace social cognition among young adults. Work experience allows young adults to learn the norms, values, and required behaviors of the business world; test their interests and skills; and build their professional and social networks (Waldeck and Myers, 2007). Obtaining real-world work experience enables students to adapt themselves to the existing work culture and eases their transition from an academic lifestyle (Pastore, 2014; Peltz et al., 2020). Work experience equips students with etiquettes that are required to thrive in the professional realm, and also prepares students to work within a multicultural environment and interact with people of all ages and social backgrounds (Peltz et al., 2020). Work experience helps students harness the right attitude and boost their self-confidence, which is vital for building a successful career (Smith et al., 2019). For these reasons, we expected that the positive association of sense of power with motivational and behavioral CQ would become stronger as an individual gains more work experience. We proposed that the relationship between sense of power and IE intention via motivational and behavioral CQ will be stronger as young adults accumulate more work experience.

H4: Work experience accentuates the mediated effects of sense of power on young adults’ IE intention through (a) motivational CQ and (b) behavioral CQ.

## Materials and Methods

### Sample and Data Collection

This study used both self-report and objective data. Self-report data were responses to a survey that was completed by senior undergraduate students (4th year) of 19 universities in China, were surveyed in 2019, 2 months prior to graduation. A total of 610 respondents, most of whom were students at Zhejiang University City College (29.9%), Zhejiang University (27.2%), and Zhejiang University of Technology (30.2%), were randomly selected and asked to complete a paper and pencil survey. Objective data included information about work experience (the number of hours per year) obtained from a survey administered to the respondents’ academic home departments. For this, we contacted the academic departments with which students were affiliated and obtained an Excel worksheet that detailed the number of hours of each respondent’s work experience. After removing incomplete surveys, a total of 372 responses were received (an effective response rate of 60.98%). Over half of respondents were female (59%), 35% were male, and 6% did not provide information on their sex. The majority (61%) were between 21 and 22 years old. Most (65%) were single, 33% were in committed relationships, and 0.8% were married.

### Measures

Our measures were translated from English to Chinese using back-translation procedures to ensure the content validity of all the measures (Glidden-Tracey and Greenwood, 1997).

### Social Class

Following prior research (Anderson et al., 2012), social class was assessed by presenting students with a picture of a 10-rung ladder. They were asked to place themselves on the ladder (check a rung) based on where their parents stood relative to other students’ parents in China in terms of income, education, and occupation. Responses were converted to a 10-point scale ranging from 1 (lowest rung; lowest perceived social class) to 10 (highest rung; highest perceived social class).

### Income

Income was assessed using Anderson et al.’s (2012) item, “Which category best describes your parents’ monthly household income?”, (a) ≤5,000 yuan, (b) 5,000–16,000 yuan, (c) 16,000–27,000 yuan, (d) 27,000–38,000 yuan, or (e) >38,000 yuan.

### Sense of Power

Sense of power was measured using Dubois et al.’s (2015) 4-item measure that assesses respondents’ subjective evaluation of being powerful and in control (e.g., “I can get people to listen to what I say”). The items were scored on a 5-point Likert scale ranging from 1 = strongly disagree to 5 = strongly agree. Cronbach’s alpha was 0.78.

### Cultural Intelligence

Cultural intelligence was measured using Van Dyne et al.’s (2008) measure, which includes a 5-item motivational CQ section (e.g., “I am confident that I can socialize with locals in a culture that is unfamiliar to me”) and a 5-item behavioral CQ section (e.g., “I am a flexible person in culturally diverse situations”). Items were scored on a 5-point Likert scale ranging from 1 = strongly disagree to 5 = strongly agree. Cronbach’s alpha was 0.76 for motivational CQ and 0.83 for behavioral CQ.

### Work Experience

Work experience was measured using a single item, “During college, how many hours per year has this student worked outside the university?” Students’ academic home departments provided this information in an Excel file.

### International Entrepreneurship Intention

International entrepreneurship intention was measured using Chandra and Coviello’s (2010) six-item measure (e.g., “I am ready to do anything to be an international entrepreneur”). Items were scored on a 5-point Likert scale ranging from 1 = a little to 5 = quite a lot. Cronbach’s alpha was 0.95.

### Control Variables

We controlled for sex because men have been found to identify international opportunities twice as often as their female counterparts (Vinogradov and Jørgensen, 2017).

Also, this study asked participants to indicate their age in years, department by creating three dummy variables: business (1 = business; 0 = art or science), art (1 = art; 0 = business or science), and science (1 = science; 0 = business or art), as these variables have been found to be associated with EI (Lanero et al., 2016).

### Common Method Variance

To explore the possibility of common method variance, we conducted a confirmatory factor analysis (Podsakoff et al., 2012) using AMOS 22.0 to test whether the hypothesized model captured distinct constructs. As can be seen in Table 1, the results of the confirmatory factor analysis indicated that the hypothesized seven-factor model was a good fit to the data [χ^2^_(129)_ = 356.587, CFI = 0.93, TLI = 0.92, RMSEA = 0.04, and SRMR = 0.05]. In addition, we compared this seven-factor model with three alternative models. The seven-factor model fitted the data better than any of the alternative models. In summary, the analyses indicated that the seven constructs captured distinctiveness, the model exhibited a theoretically meaningful structure, and the covariance structure was probably not dominated by common method variance.

**TABLE 1:** Confirmatory factor analyses.

*X* ^2^	df	CFI	TLI	RMSEA	SRMR
1. 356.587	129	0.93	0.92	0.04	0.05
2. 320.861	132	0.88	0.86	0.08	0.08
3. 366.975	134	0.85	0.83	0.08	0.08
4. 1072.981	135	0.41	0.34	0.17	0.10

*^1^Seven-factor model: income, social class, sense of power, motivational CQ, behavioral CQ, work experience, and IE intention.*

*^2^Six-factor model: combined motivational CQ and behavioral CQ.*

*^3^Five-factor model: combined motivational CQ, behavioral CQ, and IE intention.*

*^4^Four-factor model: combine sense of power, motivational CQ, behavioral CQ, and IE intention.*

### Data Analysis Techniques

Since our hypotheses involve moderated mediation, we used Hayes’ (2013) PROCESS macro 2.15 (Model-4 and 14). The effect of a first-stage moderated mediation is mathematically a linear function of the moderator; the slope of this function is a product of the coefficient of the XW on M and the coefficient of M on Y,^1^ which is also called an index of the moderated mediation (Hayes, 2015). If this index is different from zero, it leads to the conclusion that an indirect effect is moderated. We used 5,000-sample bootstrapping in this study for all the computations to yield 95% bias-corrected confidence intervals (95% CIs). If the confidence interval excludes zero, the existence of a significant effect is inferred (Hayes, 2015). Key descriptive statistics for all variables are reported in Table 2.

**TABLE 2:** Correlations and discriminant validity.

Construct	Mean	SD	1	2	3	4	5	6	7	8	9	10	11	12
1. Sex	1.34	0.56	−											
2. Age	22.65	10.61	–0.02	−										
3. Business department	0.63	0.48	0.20**	–0.07	−									
4. Art department	0.30	0.46	−0.22**	0.08	−0.87**	−								
5. Science department	0.05	0.22	0.05	–0.01	−0.30**	−0.15**	−							
6. Parents’ income (monthly)	2.29	1.35	0.14**	–0.04	0.35**	−0.39**	0.03	−						
7. Social class	5.54	1.61	–0.04	0.04	0.12*	−0.19**	0.11*	0.49**	−					
8. Sense of power	3.30	0.70	–0.08	0.04	0.10*	−0.11*	0.01	0.26**	0.20**	(0.78)				
9. Motivational CQ	3.35	0.72	–0.02	–0.03	–0.02	0.01	0.01	0.19**	0.19**	0.52**	(0.76)			
10. Behavioral CQ	3.45	0.70	−0.18**	0.04	–0.08	0.08	0.00	0.07	0.14**	0.49**	0.63**	(0.83)		
11. Work experience (hours)^a^	1.37	2.74	–0.05	0.07	0.02	–0.04	0.05	0.03	0.12*	–0.03	0.05	0.02	–	
12. IE intention	2.70	0.87	0.11*	0.00	0.22**	−0.22**	–0.00	0.38**	0.20**	0.28**	0.37**	0.12*	0.08	(0.95)

*p < 0.05, **p < 0.01. ^a^Objective data; a Reliabilities (Cronbach’s alpha); Sex: Male = 1, female = 0.

## Results

Hypothesis 1a proposed a positive relationship between SES and IE intention. As shown in Table 3, hypothesis 1a was supported; parents’ income was significantly and positively related to IE intention (effect = 0.21, 95% CI = 0.154, 0.278). Hypothesis 1b, was supported; parents’ social class was significantly and positively related to intent to remain (effect = 0.08, 95% CI = 0.028, 0.136).

**TABLE 3:** Indirect path coefficients.

Path	Direct effect		Indirect effect	
	Effect	95% BC confidence interval	Effect	95% BC confidence interval
Parents’ income → IE intention	0.21	(0.154, 0.278)		
Parents’ social class → IE intention	0.08	(0.028, 0.136)		
Parents’ income → Sense of power → IE intention			0.03	(0.014, 0.056)
Parents’ social class → Sense of power → IE intention			0.02	(0.011, 0.051)
Sense of power → Motivational CQ → IE intention			0.19	(0.105, 0.300)
Sense of power → Behavioral CQ → IE intention			–0.01	(–0.105, 0.070)

H2a and H2b were that sense of power mediates the effect of parents’ income and social class, respectively, on IE intention. As shown in Table 3, sense of power fully mediated the relationship between parents’ income and IE intention (effect = 0.03; 95% CI = 0.014, 0.056). Sense of power also fully mediated the relationship between parents’ social class and IE intention (effect = 0.02; 95% CI = 0.011, 0.051). H1a and H1b were therefore supported.

H3a and H3b were that motivational and behavioral CQ, respectively, mediate the effect of sense of power on IE intention. As shown in Table 3, motivational CQ fully mediated the relationship between sense of power and IE intention (effect = 0.19; 95% CI = 0.105, 0.300). However, behavioral CQ did not mediate the relationship between sense of power and IE intention (effect = –0.01; 95% CI = –0.105, 0.070). These results support H3a, but not H3b.

H4a was that work experience accentuates the mediated effect of sense of power on IE intention via motivational CQ. At lower levels of work experience, sense of power did not have a significant indirect effect on IE intention via motivational CQ (b = 0.19, 95% biased CI = –0.121, 0.192). However, at higher levels of work experience, sense of power did have a significant positive indirect effect on IE intention via motivational CQ (b = 0.20, 95% biased CI = 0.202, 0.582). As shown in Table 4, the index of moderated mediation was 0.034 and its 95% CI excluded zero (0.012, 0.017). Thus, H4a was supported.

**TABLE 4:** Conditional indirect effect process analysis.

Conditional effect	Level	Effect	Boot S.E.	95% BC confidence interval
Sense of power on IE intention through motivational CQ at levels of work experience	Low	0.19	0.04	(–0.121, 0.192)
	High	0.20	0.05	(0.202, 0.582)
	Index of moderated mediation	0.00	0.00	(0.012, 0.017)
Sense of power on IE intention through behavioral CQ at levels of work experience	Low	–0.01	0.04	(–0.103, 0.068)
	High	–0.01	0.04	(–0.105, 0.076)
	Index of moderated mediation	–0.00	0.00	(–0.003, 0.004)

H4b was that work experience accentuates the mediated effect of sense of power on IE intention via behavioral CQ. At lower levels of work experience, sense of power did not have a significant indirect effect on IE intention via behavioral CQ (b = –0.01, 95% biased CI = –0.103, 0.068). However, at higher levels of work experience, sense of power did have a significant positive indirect effect on IE intention via behavioral CQ (b = –0.01, 95% biased CI = 0.105, 0.076). As shown in Table 4, the index of moderated mediation was 0.044 and its 95% CI excluded zero (–0.003, 0.004). H4b was not supported.

### Ancillary Analyses

To further investigate the moderating effect of work experiences, we used the Johnson-Neyman technique (Preacher et al., 2007). This technique determines the region of significance for the conditional indirect effect of a moderator mathematically. This technique offers a more complete picture than the more common pick-a-point approach (i.e., simple-slopes analysis or spotlight analysis), especially when the moderator is a continuous variable (Preacher et al., 2007).

**FIGURE 2:**
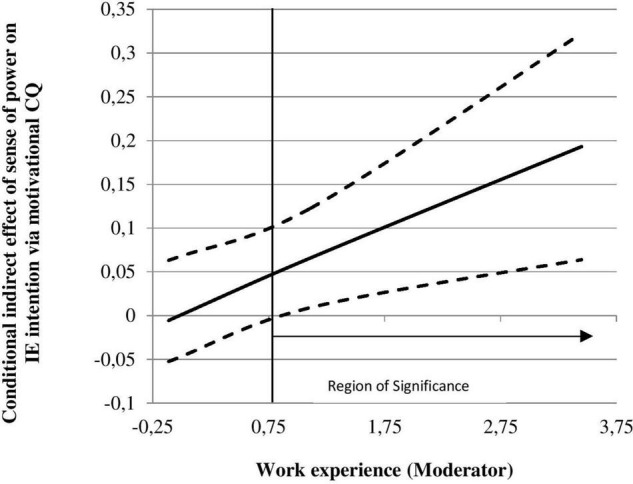
Johnson-Neyman regions of significance for the H4a moderated mediation. The solid line depicts the trajectory of the conditional indirect effect, and the dashed lines depict the upper and lower limits of the 95% CI.

As shown in Figure 2, the mediated effect of sense of power on IE intention via motivational CQ was non-significant at work experience values below 0.75, but for values above this, the mediated effect became positive and significant, and gradually increased. These results are consistent with the predictions of H3a, but not for H3b.

To gain further insights about the underlying psychological mechanism that links parents’ SES to IE intention, we also tested the serial mediation (parents’ SES→sense of power→CQ→IE intention) using the bias-corrected bootstrap analysis of PROCESS model #6 (see Table 5). Using parents’ income as the X variable, the serial mediation was significant via motivational CQ (SE = 0.024; 95% CI = 0.011, 0.041), but non-significant via behavioral CQ (SE = 0.000; 95% CI = –0.011, 0.012). Using social class as the X variable, the serial mediation was significant via motivational CQ (SE = 0.016; 95% CI = 0.006, 0.030), but non-significant via behavioral CQ (SE = –0.001; 95% CI = 1.010, 0.005).

**TABLE 5:** Serial mediation test in PROCESS macro Model 6 (5,000 bootstrap samples at 95% confidence).

	Direct effect						Indirect effect									Total
	a	b	c	d	e	f	g = b*e			h = a*f			i = b*c*f			d+g+h+j
	X→M1	X→M2	M1→M2	X→Y	M1→Y	M2→Y	X→M1→Y			X→M2→Y			X→M1→M2→Y			X→Y
	β	β	β	β	β	β	Est.	Lo	Hi	Est.	Lo	Hi	Est.	Lo	Hi	Est.
PI→SP–MCQ→IEI	**0.138**	**0.030**	**0.523**	**0.205**	**0.067**	**0.339**	0.009	–0.013	0.032	0.010	–0.004	0.030	**0.024**	0.011	0.041	**0.250**
PI→SP–BCQ→IEI	**0.138**	**–0.034**	**0.512**	**0.216**	**0.240**	0.010	**0.033**	0.010	0.060	–0.000	–0.006	0.007	0.000	–0.011	0.012	**0.250**
PSC→SP–MCQ→IEI	**0.090**	**0.042**	**0.516**	**0.069**	**0.135**	**0.349**	0.012	–0.002	0.031	**0.014**	0.000	0.033	**0.016**	0.006	0.030	**0.112**
PSC→SP–BCQ→IEI	**0.090**	**0.017**	**0.486**	**0.084**	**0.332**	**–0.033**	**0.030**	0.010	0.056	–0.000	–0.005	0.005	–0.001	–0.010	0.005	**0.112**

*Effect sizes in bold are sig: P < 0.001 for effects > 0.089; p < 0.01 for effects > 0.060; p < 0.05 for effects > 0.013. PI, parents’ income; PSC, parents’ social class; SP, sense of power; MCQ, motivational CQ; BCQ, behavioral CQ; IEI, IE intention.*

*^1^X/Y refers to independent/dependent variable, while M/W means mediator/moderator, respectively.*

## Discussion

The main purpose of this study was to use the Social Cognitive Theory of Social Class (Bandura, 1989; Kraus and Stephens, 2012) to extend our understanding of the factors that influence young adults’ intention to become an international entrepreneur.

### Theoretical Implications

First, the two dimensions of parents’ income and social class salience confirmed the Social Cognitive Theory of Parent’s SES (Adler et al., 2000; Kraus and Keltner, 2013). One explanation as to why career ambitions differ between individuals is that people from different SESs develop different social cognitive tendencies. Our results corroborate this view; we found greater levels of resources and superior career choices among students from high-SES families relative to those from low-SES families. Specifically, Social Cognitive Theory helps predict IE intention using SES differences (parents’ material resources and social class); these differences engender interpersonal differences in terms of social cognitive tendencies, which in turn influence behavioral intentions (Kraus et al., 2012).

The present study demonstrated that parents’ SES influences motivational CQ and IE intention via an individual’s sense of power. Previous research has demonstrated that one’s sense of power flourishes to a greater extent when socioeconomic needs are anticipated and met by parents (Kraus et al., 2012). The current study thus provides further evidence in support of this proposition; however, more importantly, we identified motivational CQ and IE intention as two positive outcomes of sense of power. Sense of power is the psychological bridge between parents’ SES and motivational CQ because the greater the feeling of self-empowerment that accompanies being supported by parents’ resources, the more capable an individual is of functioning effectively in a cross-cultural environment. Sense of power fosters motivational CQ by increasing interest, intrinsic motivation, self-efficacy, and confidence in dealing with cross-cultural encounters (Ang et al., 2015). Moreover, power is an essential prerequisite and attribute for entrepreneurship [e.g., Begley and Boyd (1987)], and so our finding of an impact of sense of power on IE intention via motivational CQ provides both theoretical and empirical support.

Third, motivational CQ emerged as the second leg in the salient moderated serial mediation of the effect of parents’ SES on IE intention. Specifically, motivational CQ increased as young adults felt an increased sense of power; with a higher motivational CQ, they were more likely to consider IE. While prior research has acknowledged CQ as a mitigator of IE’s inherent uncertainty (Ang et al., 2007), it has not specified the specific role of CQ as a mediator. This study conceptualizes and empirically supports the view that motivational CQ is the second leg of the psychological process that serially mediates the effect of parents’ SES on IE intention. Moreover, this study identified motivational CQ as the only relevant CQ domain, in that it was the only one to predict IE intention. Behavioral CQ failed to emerge as a significant underlying mechanism of this relationship.

Finally, we found that the mediated effect of sense of power on IE intention was stronger when young adults had had work experience, whether self-employed or working for others. As expected, the mediated effect of sense of power on IE intention via motivational CQ was non-significant at low levels of work experience, but positive and significant at moderate and high levels of work experience. Although previous studies have established that parents’ prior self-employment experience increases the likelihood of an individual’s entrepreneurial career choices (Nguyen, 2018), the present study extends the literature by examining the moderating effect of the individual’s own work experience.

### Practical Implications

Our findings have practical implications that could help to enhance young adults’ IE intention. First, parents, educators, business people, and policy makers should remain up-to-date on the effect of parents’ SES on young adults’ psychological conditions and behavioral responses. Although it is naïve to assume that parents and/or policy makers can make significant improvements to their SES quickly and across the board, simply being aware of the salient effect of SES could serve as a reminder that many of the differences observable across young adults are rooted in socioeconomic hierarchies, i.e., the realities that affect young adults discriminately. Being mindful of the structural determinants of career ambitions, such as IE intention, is prerequisite to address the discrepancies in SES and enhance IE intentions at the familial and societal levels.

Another practical implication pertains to the psychological mechanism underlying the effect of parents’ SES and IE intention. Knowing that IE intentions are predicated on factors such as sense of power and motivational CQ, this resonates with the need to continually seek ways to enhance young adults’ cognitive, affective, and behavioral qualities prior to and during the crucial transition period from school to work. Ambitious career intentions such as IE require an individual to realize and believe in the power within, and to have an intrinsic interest in cross-cultural interactions. Given the lack of initiatives that can sustainably alter the factor of parents’ SES, parents, educators, businesspeople, and policy makers should identify alternative activities that could help to enhance young adults’ feeling of self-empowerment and boost their motivational CQ. Examples of such activities include providing cross-cultural and international learning opportunities, competitions, and internships; expanding foreign language programs and curricula; establishing intercultural student organizations that hold international workshops and forums; and, most importantly, motivating young adults from all familial backgrounds to take advantage of such resources. These experiences may encourage the state of mind required to become more appreciative of and interested in IE.

While, these resources and initiatives could be instrumental in expanding young adults’ socio-cognitive capacities, they do not replace or remove the need for real-world work experience. Our results suggest that work experience accentuates the positive influence of motivational CQ on IE intention. Therefore, enabling and promoting cross-culturally relevant work experience is another avenue to pursue as part of a concerted effort to cultivate IE intention.

In final, in 2013, China’s President Xi Jinping proposed the Belt-and-Road Initiative (BRI) to encourage economic partnerships between China and other nations (Wu and Ruan, 2018). As a result, the Chinese government began issuing a series of policies and measures that have been adopted by Chinese universities to encourage college students to start their own businesses. Therefore, our research model can be a guideline to help Chinese universities to produce high-quality talent entrepreneur to meet the current or future demand created by the BRI (Budhwar et al., 2017; Su et al., 2020).

### Limitations and Future Research

This study has three major limitations. First, it was a cross-sectional study, which did not allow us to capture the effects of SES on sense of power, motivational CQ, and IE intention at different points in time. Future research could adopt longitudinal methods to complement and extend our findings. The use of longitudinal designs will reduce the potential for CMV. Although steps (e.g., use of well-established validated scales) were taken in the design and implementation of this study to reduce CMV (Podsakoff et al., 2012) and the Harmon test results suggest it was not an issue, its effects cannot be completely ruled out.

Second, this study only took the first period before graduation into account. While, this first period clearly is important in the students’ IE intention [e.g., Velinov et al. (2020)], in the future studies a longer time span may need to provide for additional insight. For example, early-IE will face multiple challenges (e.g., cultural differences and language barriers) after the transition to the overseas market (Sinkovics et al., 2004; Cumming and Zhan, 2018). Therefore, in line with other research on the transition to the overseas market, our suggestion for the future studies that needs to be to follow up individuals after three and five years at the overseas market [e.g., Giacomin et al. (2011)].

Third, this study focused on the IE intentions of young adults. Future studies could assess the external validity of our proposed model by (a) applying it to other segments of the population and (b) using the independent and mediator variables to explain entrepreneurship intentions in general, as well as other career choices in the public or private spheres.

Fourth, some studies have suggested that the relationship between parents’ SES and young adults’ career decisions is weaker in developing countries’ economically challenged context relative to economically advanced developed countries (OECD, 2019). Thus, further research is needed to elucidate the external validity of our proposed model across political and cultural boundaries. Indeed, scholars have called for comparative studies of the effect of familial background across Western and Eastern cultures.

Finally, future research could explore other factors worthy of inclusion in the nomological network of constructs in the form of antecedent, mediator, or moderator variables. Given that young adults are an important element of the global expansion strategies of the Belt and Road Project; we hope that future research will further investigate the present findings.

## Data Availability Statement

The raw data supporting the conclusions of this article will be made available by the authors, without undue reservation.

## Ethics Statement

The study was reviewed and approved by the School of Business of the Zhejiang City College University’s Ethics Committees Professor Wenwu Xie and Jianzhuang Zheng. The patients/participants provided their written informed consent to participate in this study.

## Consent to Participate

Every participant consent was obtained after they were provided information on the “aims, methods, duration of the questionnaires, sources of funding, any possible conflicts of interest, institutional affiliations of the researcher, the anticipated benefits and potential risks of the study and the discomfort it may entail”; between information and consent stage we gave every participant at least 48 h to think about whether to consent or not. Moreover, we confirmed that our research was conducted in an independent and unbiased manner.

## Author Contributions

MJ: data collection, theory development, writing, and analyzing.

## Conflict of Interest

The author declares that the research was conducted in the absence of any commercial or financial relationships that could be construed as a potential conflict of interest.

## Publisher’s Note

All claims expressed in this article are solely those of the authors and do not necessarily represent those of their affiliated organizations, or those of the publisher, the editors and the reviewers. Any product that may be evaluated in this article, or claim that may be made by its manufacturer, is not guaranteed or endorsed by the publisher.
